# hsa_circ_0007841: A Novel Potential Biomarker and Drug Resistance for Multiple Myeloma

**DOI:** 10.3389/fonc.2019.01261

**Published:** 2019-11-19

**Authors:** Meng Gao, Chengyuan Li, Han Xiao, Hang Dong, Siyi Jiang, Yunfeng Fu, Liying Gong

**Affiliations:** The Third Xiangya Hospital of Central South University, Changsha, China

**Keywords:** circular RNAs, biomarker, diagnosis, prognosis, multiple myeloma

## Abstract

**Purpose:** Circular RNA (circRNA) is a key regulatory factor in the development and progression of human tumors. However, the working mechanism and clinical significance of most circRNAs remain unknown in human cancers, including multiple myeloma (MM).

**Patients and Methods:** This study employs high-throughput circRNA microarray with bioinformatics to identify differentially expressed circRNAs in patients with MM. The hsa_circ_0007841 expressions were observed in the MM tissues of 86 patients. Drug-resistant cell lines and pathological features were also detected. In addition, the relationship between hsa_circ_0007841 expressions in the MM tissues and the pathological features of patients with MM were evaluated and role of hsa_circ_0007841 as a potential biomarker and therapeutic target was assessed.

**Results:** The results show that in the MM cell lines and drug-resistant cell lines, hsa_circ_0007841 expression was significantly upregulated, which was closely associated with disease prognosis. Specifically, hsa_circ_0007841 upregulation was correlated with chromosomal aberrations such as gain 1q21, *t* (4:14) and mutations in ATR and IRF4 genes. This finding was corroborated in large samples. Finally, bioinformatics analysis showed that eight differentially expressed miRNAs and 10 candidate mRNAs interacted with hsa_circ_0007841, shedding some new light on the basic functional research.

**Conclusion:** This study may be the first to report that hsa_circ_0007841 is significantly upregulated in MM. It also suggests that hsa_circ_0007841 may be a novel biomarker for MM and its involvement in the progression of MM.

## Introduction

Non-coding RNA (ncRNA) consists of long non-coding RNA (lncRNA), short microRNA (miRNA/miRs), and circular RNA (circRNA), the latter being the most common in the eukaryotic transcriptome. Unlike the conventional linear RNA (including 5′ and 3′ terminals), circRNA has a closed loop structure generated by back-splicing of RNA introns and/or exons. After the discovery of circRNA in viral RNAs in 1970 (1), it was later found in eukaryocytes (2). In recent years, high-throughput sequencing combined with transcriptome analysis has indicated the large abundance of circRNA in eukaryocytes (3).

Circular RNAs, repleted with miRNA binding sites, serve as sponge molecules for miRNAs and inhibit the binding of miRNAs to their target genes (4, 5). They bind competitively to miRNAs through the sponging action, thereby abolishing the inhibitory effect on the transcription of downstream target genes (6). Besides sponging action, circRNAs can also regulate gene expressions in parents and influence gene transcription or even protein translation by interacting with the proteins. Moreover, circRNAs are large RNA molecules with closed loop structures. Most ncRNAs and circRNAs are involved in the regulation of transcription and posttranscriptional gene expression. They play important roles in cancer progression, metastasis, and therapeutic response (7). Given the specificity of circRNAs in the disease state and their stability in the body fluid, circRNAs may be used in the diagnosis of cancer (8, 9).

Multiple myeloma (MM) is a hematologic malignancy caused by an abnormal proliferation of bone marrow plasma cells. In the clinical setting, MM is the second most common hematologic malignancy and accounts for 10% of all hematologic malignancies. Its incidence rate reaches 2–3 per 100,000. The ratio of affected male to female patients is 1.6:1. The features of MM are diverse but include anemia, bone pain, renal insufficiency, infection, hemorrhage, neurological symptoms, hypercalcemia, and amyloidosis (10). The development of MM is usually accompanied by a series of genetic variations, such as cytogenetic abnormalities, primary or secondary chromosomal translocation, and some oncogenes activations (11). Identifying these alterations is highly valuable for understanding the pathogenesis of tumors and predicting prognosis and therapeutic response. Some genetic mutations are correlated with the poor outcome of MM, including chromosomal variations *t* (11:14), *t* (14:16), and *t* (14:20) (12). The identified mutations include CCND1 and DNA repair pathway-related genes (TP53, ATM, ATR, and ZFHX4) (13). In contrast, some mutations predict positive outcomes such as mutations in the IRF4 and EGR1 genes (14). Individualized therapy for MM based on biomarkers can increase therapeutic efficacy while reducing toxicity (15). Therefore, some biomarkers for MM can be used as predictive and prognostic indicators to guide diagnosis and treatment.

In the present study, the bioinformatics method was combined with high-throughput sequencing in small samples. By using the circRNA database, circRNAs that might influence the treatment and prognosis of patients with MM were preliminarily screened. Next, real-time quantitative polymerase chain reaction (qRT-PCR) was applied for sample amplification. It was found that hsa_circ_0007841 was significantly upregulated in patients with MM and MM cell lines. The correlation between hsa_circ_0007841 expressions and clinicopathological features of such patients with MM was determined. It was determined whether the hsa_circ_0007841 expression could be used as a diagnostic and prognostic indicator for MM. The results provide the basis for identifying novel prognostic markers and therapeutic targets for MM.

## Materials and Methods

### Clinical Data

From January 2012 to January 2018, 86 MM patients and 30 IDA patients treated at the Third Xiangya Hospital of Central South University were included as the case group. Their bone marrow samples and clinical data were collected. There were 53 males and 33 females in the case group, and the median age of onset was 55 years (range, 44–78 years). The diagnosis, staging, and risk stratification of MM were performed according to the National Comprehensive Cancer Network (NCCN). All of the cases had complete clinical and pathological data (Table S1). Due to a lack of normal bone marrow donors, 30 patients with iron deficiency anemia (IDA) were chosen as controls, and their bone marrow samples were collected to avoid sample variation. The bone marrow samples were cryopreserved at −80°C. The collection of all samples was approved by the ethics committee of Xiangya third hospital (Approval number: 2016121), and the consent acquired was both written and informed consent.

The microarray data has been deposited in public, community-supported repositories (GEO, GSE133058).

### Cell Culture

Normal human mononuclear cell line THP-1 and MM cell lines KM3, U266, and RPMI-8226 were provided by the basic laboratory of Central South University Xiangya School of Medicine. Drug-resistant cell lines KM3/BTZ, U266/BTZ, and RPMI-8226/BTZ were acquired by inducing tolerance through stepwise increase of drug concentrations. The cells were cultured in the 1,640 culture medium (HyClone, Logan, UT, USA) supplemented with 10% fetal bovine serum (ExCell Biology, Shanghai, China), 50 U/ml penicillin, and 50 g/ml streptomycin (HyClone). The cells were incubated in a 37°C, 5% CO_2_ incubator and harvested in the log phase of growth.

### RNA Extraction

Firstly, the bone marrow samples from 3 MM patients were sorted by magnetic activated cell sorting (MACS) using anti-CD138 MicroBeads (Miltenyi, Germany), and the plasma cells were enriched. According to the instruction manual of TRIzol reagent (Invitrogen, USA), total RNA extraction was performed from the enriched plasma cells and plasma cells in the normal human bone marrows. The extracted products were preserved at −80°C. The RNA concentration and activity were first detected by NanoDrop ND-1000 (NanoDrop, USA). Then RNA purity and integrity were detected by denaturing formaldehyde agarose gel electrophoresis.

### High-Throughput Sequencing of circRNAs and Identification of Differentially Expressed circRNAs

Sample preparation and microarray hybridization were performed according to Arraystar's standard protocols. First, linear RNAs in the extracted total RNA were removed by using RNaseR (Epicentre, USA), and the circRNAs were enriched. Then, using random primers, the enriched circRNAs were amplified and transcribed into fluorescent cRNAs. Alignment and hybridization were performed between the labeled circRNAs and Arraystar Human circRNA Array V2 (8x15K, Arraystar), followed by washes with the cleaning solution kit (Agilent, USA). The microarrays were scanned with the Agilent Scanner G2505C. The microarray images were analyzed with Agilent Feature Extraction software (version 11.0.1.1). The quantiles were normalized and subsequent data processing was performed using limma package in R. Differentially expressed circRNAs were identified based on the fold change and *P* value. Significantly upregulated and downregulated genes were those with fold change > 2.0 and *P* < 0.05.

### Verification of Quantitative RT-PCR

Sample RNA was reversely transcribed into cDNA using SuperScript III Reverse Transcriptase (Invitrogen, Grand Island, NY, USA). Real-time qRT-PCR (Arraystar) was implemented by using ViiA 7 Real-time PCR System (Applied Biosystems) and 2 × PCR Master Mix. Each mix (10 μL) contains 2 × Master Mix 5 μL, specific primer F 0.5 μL, specific primer R 0.5 μL, and 2 μL cDNA. The mix was added into each well of the 384-Well PCR Plate, which was then placed in the real-time PCR instrument for PCR reaction. The reaction conditions were as follows: incubation at 95°C for 10 min, then 40 cycles of 10 s at 95°C and 1 min at 60°C. GAPDH was used as internal reference and its expression was normalized. ΔCt value reflected the circRNA expression level. The expression levels of each target gene were calculated and normalized by that of the internal reference. The primers used for each target gene are shown in Table 1.

**Table 1:** Primers designed for qRT-PCR validation of candidate circRNAs.

	**Primer**	**Tm (^**°**^C)**
β-actin (human)	F: 5′ GTGGCCGAGGACTTTGATTG 3′	60
	R: 5′ CCTGTAACAACGCATCTCATATT 3′	
hsa_circ_0010402	F: 5′ ACAAATCTGGCTCTGGCACC 3′	60
	R: 5′ GAGGTAGACAGATGAGACGCCA 3′	
hsa_circ_0004777	F: 5′ CTGGACTTCAACCGAAATTTGA 3′	60
	R: 5′ GCTCCTCATGGACATCCTTTG 3′	
hsa_circ_0007841	F: 5′ CTAACATCTGTGAAACCATCGT 3′	60
	R: 5′ TCATCACATACACGATAGACTGG 3′	
hsa_circ_0080212	F: 5′ CTACCTGAGCCAGTTCTCCTAA 3′	60
	R: 5′ GGGTTCCTCATCGGTGTAAT 3′	

### Functional Analysis of circRNA

The circRNA/microRNA interaction was predicted using Arraystar's home-made miRNA target prediction software based on miRanda and TargetScan. To establish circRNA-miRNA network, we searched MREs on circRNAs using the software, then selected the miRNAs according to seed match sequences. The graph of the circRNA/miRNA network was drawn with the help of Cytoscape 3.01 (16, 17). GO analysis was conducted using Kyoto Encyclopedia of Genes and Genomes GO analysis was also used to determine the biological functions of the adjacent protein-coding genes of the target circRNAs (18).

### Statistical Analysis

SPSS 20.0 software was used for statistical analysis. The data were described as mean ± standard deviation. Mann-Whitney test was used for pair wise comparison, and Kruskal-Wallis *H*-test for multiple comparison. For the analysis of clinical data, Kaplan-Meier survival curve was plotted, and log-rank test was used for statistical hypothesis test. The correlation between circRNA expressions and chromosomal variation and genetic mutations was analyzed by the chi-squared test. *P* < 0.05 indicated significant difference.

## Results

### Analysis of circRNA Profiles

**Figure 1:**
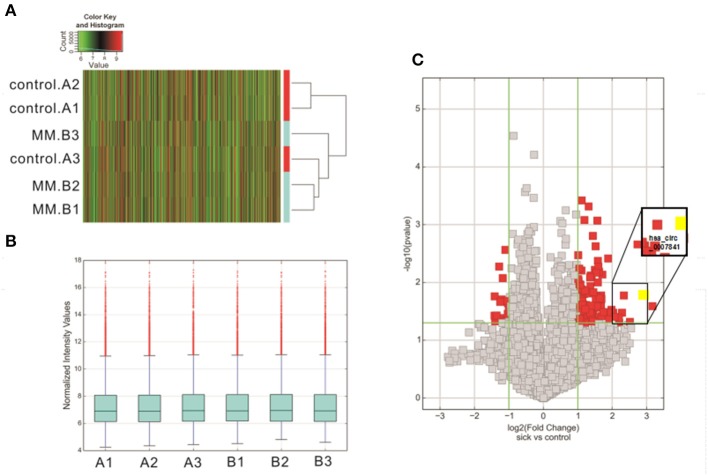
Overview of the microarray signatures. **(A)** Cluster analysis diagram of differentially expressed circRNAs. **(B)** The block diagram represents the normalized intensity of the two groups. **(C)** Volcano plot showing the significantly deregulated genes in MM samples. MM, multiple myeloma.

To identify the differentially expressed circRNAs in MM patients, high-throughput circRNA microarray was applied to 3 MM patients and 3 IDA patients. Thousands of circRNAs were detected, including 4,727 upregulated circRNAs and 5,283 downregulated circRNAs (Figure 1A). There were 147 differentially expressed circRNAs (fold change > 2), including 131 upregulated and 16 downregulated circRNAs. The block diagram represents the normalized intensity of the two groups (Figure 1B), and the volcano plot indicates the differential expressions of circRNAs (Figure 1C).

### hsa_circ_0007841 Was Upregulated in MM Cells

**Figure 2:**
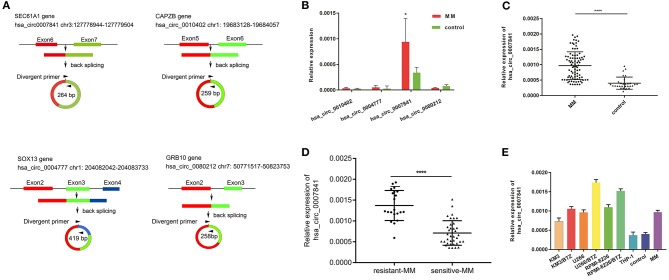
Differentially expressed circRNAs. **(A)** Key circRNAs are circulated by exons. **(B)** qRT-PCR verification of four circRNAs in MM patients and controls. **(C)** Expression of hsa_circ_0007841 in the bone marrow of 86 MM patients and 30 controls. **(D)** Expression of hsa_circ_0007841 in bortezomib (BTZ) sensitive (*N* = 36) and bortezomib (BTZ) resistant (*N* = 21) patients. **(E)** Expression of hsa_circ_0007841 in MM patients, cell lines, and controls. MM, multiple myeloma; qRT-PCR, Real-time quantitative polymerase chain reaction. *P < 0.05, ****P < 0.01.

Four significantly upregulated circRNAs were then verified by qRT-PCR in 20 MM patients and 10 IDA patients. As shown in Figure 2B, the expression of hsa_circ_0007841 was upregulated to the highest degree. hsa_circ_0007841 is localized to chr3:127778944-127779504 and generated by back-splicing of exons 6 and 7 in the sec61a1 gene (Figure 2A). We further detected expressions of hsa_circ_0007841 in the bone marrow of 66 MM patients and 20 IDA patients (Figure 2C). The results showed that hsa_circ_0007841 may be a potential biomarker for MM. Moreover, hsa_circ_0007841 expression was also detected in THP-1 mononuclear cells, MM cell lines (KM3, U266, 8226), drug-resistant cell lines (KM3/BTZ, U266/BTZ, 8226/BTZ) (Figure 2E) and patients with MM with BTZ resistance (Figure 2D). hsa_circ_0007841 was selectively expressed in the MM cell lines but lowly expressed in the THP-1 mononuclear cells, and the difference was of statistical significance (*P* < 0.05). In bortezomib-resistant cell lines (KM3/BTZ, U266/BTZ), the expression of hsa_circ_0007841 was significantly higher than that in KM3 and U266 cells. And also significantly increased in patients with MM with bortezomib (BTZ) resistant (*N* = 21) than bortezomib (BTZ) sensitive (*N* = 36). The above results indicated that the upregulation of hsa_circ_0007841 may be involved in the bortezomib tolerance of MM patients.

### Correlation Between hsa_circ_0007841 Expressions and Clinicopathological Parameters of MM Patients

According to the 2018 NCCN Guidelines, the correlation between hsa_circ_0007841 expressions and age, gender, type, staging, and risk stratification (IMWG) of MM patients was analyzed. The clinical correlation between hsa_circ_0007841 and MM was established. The results showed (Table 2) that hsa_circ_0007841 was correlated with typing (*P* = 0.002), cytogenetic mutation (*P* = 0.025), bone destruction (*P* = 0.014), R-ISS staging (*P* < 0.001), but not correlated with gender, age, percentage of plasma cells in the bone marrow and kidney injury.

**Table 2:** Correlations between the relative expression of hsa_circ_0007841 and clinicopathologic features in 86 MM patients.

**Clinicopathologic features**	**Cases**	**hsa_circ_000784 relative expression (x ± s) × 10^**−4**^**	***P*-value**
Age (year)			0.524
<60	48	9.895 ± 4.713	
≥60	38	9.453 ± 4.259	
Sex			0.289
M	53	10.118 ± 4.615	
F	33	9.060 ± 4.300	
Isotype			0.002^*^
lgG	43	9.995 ± 4.763	
lgA	22	11.972 ± 4.047	
Light chain	12	7.147 ± 2.801	
Unclassified	9	6.144 ± 1.840	
Cytogenetic abnormality			0.025^*^
Yes	57	10.428 ± 4.520	
No	29	8.269 ± 4.166	
Percentage of myeloma cells in BM			0.655
≥40%	29	9.521 ± 4.460	
<40%	57	10.052 ± 4.630	
Bone lesion			0.014^*^
Yes	79	10.043 ± 4.516	
No	7	5.826 ± 1.553	
Renal inadequacy			0.583
Yes	19	5.582 ± 4.564	
No	67	10.113 ± 4.348	
Revised International Staging System			0.000^*^
Stage 1	10	5.569 ± 1.457	
Stage 2	37	8.087 ± 3.719	
Stage 3	39	12.290 ± 4.211	

MM, multiple myeloma; SD, standard deviation;

* *P < 0.05*.

### The Role of hsa_circ_0007841 in the Diagnosis of MM

**Figure 3:**
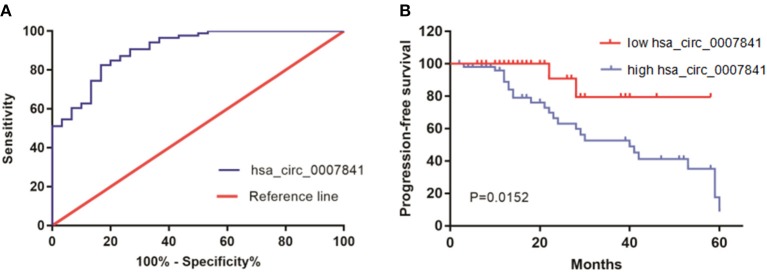
The role of hsa_circ_0007841. **(A)** ROC curve for hsa_circ_0007841. **(B)** Kaplan-Meier survival curve for low and high expression of hsa_circ_0007841 in MM patients. MM, multiple myeloma; ROC curve, receiver operating characteristic curve.

ROC curves can reflect the specificity and sensitivity of continuous variables comprehensively. ROC curves were plotted for the MM group and control group to assess the diagnostic potential of hsa_circ_0007841 in MM patients (Figure 3A). The sensitivity and specificity of hsa_circ_0007841 were verified by this method, and the AUC value was calculated as 0.907 (95% CI 0.8476–0.9663). Therefore, hsa_circ_0007841 may be used as a diagnostic marker for MM.

### Prognostic Value of hsa_circ_0007841 in MM

The survival curve was constructed to analyze the correlation between hsa_circ_0007841 and the prognosis of MM patients (Figure 3B). All 86 patients adhered to the follow-ups. Using the median hsa_circ_0007841 expression in the bone marrow of MM patients as threshold, all 86 patients were divided into low and high hsa_circ_0007841 expression groups. The prognostic value of hsa_circ_0007841 in MM patients was assessed based on progression-free survival (PFS). The survival analysis showed that the upregulation of hsa_circ_0007841 was significantly correlated with poor prognosis of MM patients (log-rank *P* = 0.0206).

Prognostic biomarkers can be used to predict the possibility of recurrence and patient survival. As the prognosis of MM patients is closely related to genetic mutations, the correlation between hsa_circ_0007841 expressions in bone marrow and cytogenetic mutation was analyzed. It was found that the upregulation of hsa_circ_0007841 was correlated with chromosomal variations gain 1q21 (*P* = 0.039) and *t* (4;14) (*P* = 0.025) and mutations in ATR and IRF4 genes, but not correlated with del (17p) or mutations in TP53 (Table 3).

**Table 3:** Cytogenetic aberration status distribution between low/high hsa_circ_0007841 expression groups of MM patients.

**Group**	**Low hsa_circ_0007841**	**High hsa_circ_0007841**	***P*-value**
del (17p)			0.721
Positive	30.2% (4/32)	53.5% (5/54)	
Negative	69.8% (28/32)	46.5% (49/54)	
gain 1q21			0.039^*^
Positive	34.4% (11/32)	57.4% (31/54)	
Negative	65.6% (21/32)	32.6% (23/54)	
*t* (4;14)			0.025^*^
Positive	6.3% (2/32)	25.9% (14/54)	
Negative	93.7% (30/32)	74.1% (40/54)	
TP53 variants			0.147
Positive	41.9% (1/32)	32.6% (8/54)	
Negative	58.1% (31/32)	67.4% (46/54)	
ATR abnormalities			0.048^*^
Positive	3.1% (1/32)	18.5% (10/54)	
Negative	96.9% (31/32)	81.5% (44/54)	
IRF4 variants			0.042^*^
Positive	6.3% (2/32)	24.1% (13/54)	
Negative	93.7% (30/32)	75.9% (41/54)	

MM, multiple myeloma;

* *P < 0.05*.

### Prediction and Annotation of Target miRNA and mRNA Networks of hsa_circ_0007841

**Figure 4:**
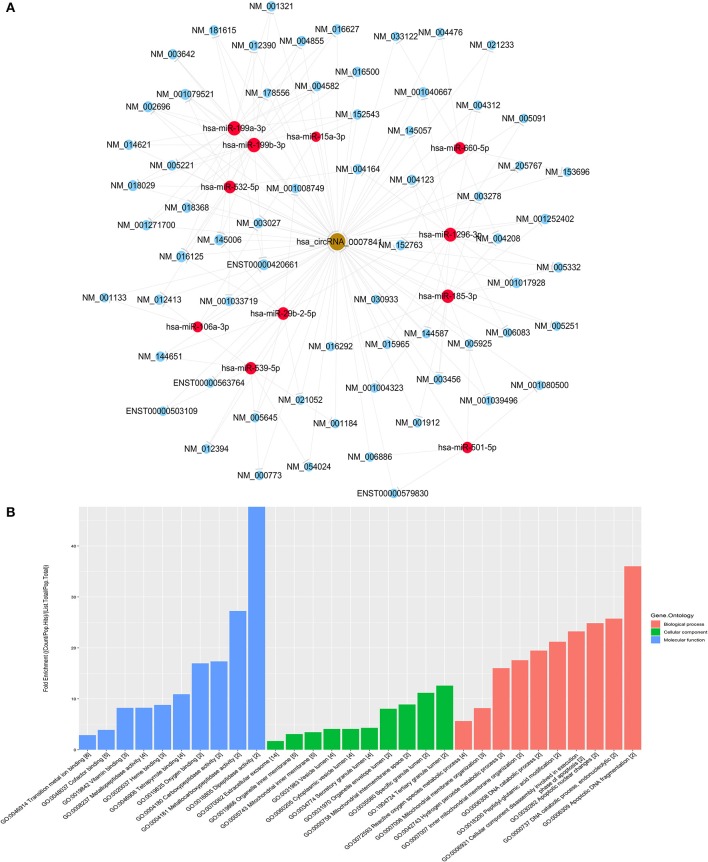
Functional analysis of circRNA. **(A)** hsa_circ_0007841-miRNA-mRNA co-expression network established using hsa_circ_0007841. **(B)** The GeneOntology_Fold Enrichment.

miRNA target prediction software TargetScan and miRanda from Arraystar was used to predict the circRNA—miRNA—mRNA network. The miRNAs and candidate mRNAs binding to hsa_circ_220 were identified. The circRNA-miRNA interaction network was predicted on the CircInteractome database (Figure 4A). A total of 8 differentially expressed miRNAs and 10 candidate mRNAs were predicted to interact with hsa_circ_0007841. As shown in Figure 4B, the predicted mRNAs might be involved in multiple pathways. Therefore, it was necessary to further investigate the role of hsa_circ_0007841 in the progression of MM.

## Discussion

The incidence of MM has been rising yearly due, in part, to the aging population. Although new therapies have greatly improved the prognosis of MM, it remains incurable. One of the major contributing factors to this is the high heterogeneity of MM cells that leads to disease recurrence and drug resistance in patients (19). Individualized therapy based on biomarkers can maximize the efficacy and reduce disease recurrence as well as drug resistance. Some of the biomarkers already identified for MM may be useful in the selection of the most suitable therapy to alter the clinical outcome (20).

circRNA is the sponge molecule for miRNA that indirectly regulates the expression of miRNA target genes; thus, playing an important role in the onset and development of human diseases. As biomarkers, circRNAs are now widely used for disease diagnosis. Recently, circRNAs have received an increasing amount of attention in the biomedical literature. Vo et al. analyzed over 2,000 human tumor specimens derived from different tissues by exome capture and studied the circRNA profiles (21). Boutros and He systematically analyzed the expression characteristics of circRNAs in localized prostate cancer and designed the shRNA library for high-throughput sequencing. They reported that circCSNK1G3 competitively bind to miR-181b/d and exhibit fundamental function on four prostate cancer cells (22). Shen et al. reported the upregulation of circFAT1, formed by exon 2 in the FAT1 gene, in human osteosarcoma (OS). Their study demonstrated the oncogenicity of circFAT1 in OS and its potential value as a therapeutic target in OS (23). In addition, specifically expressed circRNAs have been identified in hepatocellular carcinoma (24), colorectal cancer (25), and lung cancer (26). These observations suggest the idea that circRNAs may be used as potential diagnostic and prognostic biomarkers. However, the clinical value of circRNAs in MM remains unclear.

We screen carefully the key circRNA based on the following considerations: (1) the significant difference in FC (fold change) value will be considered priority; (2) each sample has the original signal value, the tendency to select the original signal value is relatively large, and the average within the group is stable; (3) small *P* value and combined with the original signal value average within the group; (4) the appropriate length is recommended within 200–2,500 bp, in order to facilitate the later functional test; (5) try to choose the exonic type. In the present study, high-throughput sequencing was used to identify differentially expressed circRNAs in the bone marrow tissues of patients with MM. Several circRNAs were found to be upregulated, four significantly upregulated circRNAs were then verified by qRT-PCR in small clinical sample and MM cell lines, include hsa_circ_0010402, hsa_circ_0004777, hsa_circ_0080212, hsa_circ_0007841, and the hsa_circ_0007841 expression showed the most significant increase. This result was later verified by qRT-PCR in large samples (*n* = 86). To the best of our knowledge, this finding is the first confirmation of the expression, diagnostic, and prognostic value of hsa_circ_0007841 in MM. By combining these parameters with the clinicopathological indicators of patients with MM, we found a significant difference in hsa_circ_0007841 expressions among patients with different typing and staging. We further analyzed disease progression during the survival period between 1 and 4 years. We found that hsa_circ_0007841 overexpression was correlated with poor prognosis in patients with MM. Moreover, since the circ-miRNA axis has a considerable impact on the onset and development of human diseases, it has been speculated that hsa_circ_0007841 might influence the occurrence of MM by mediating the expression of oncogenes through its miRNA target. Annotation and functional prediction have shown that hsa_circ_0007841 interacted with eight miRNAs and 10 target mRNAs. Among these, hsa_circ_0007841 is the sponge molecule of hsa-miR-29b-2-5p. Rossi et al. found that miR29b overexpression inhibited osteoclast differentiation and reversed the MM cells-triggered osteoclast activation, which delayed the progression of MM. Furthermore, miR-29b caused the apoptosis of the BTZ-induced MM cells through activation of the feedback loop of transcription factor Sp1 (27). In the present study, hsa_circ_0007841 overexpression was found to be correlated with osteolytic bone destruction in MM and it was overexpressed in BTZ-resistance cell lines in MM. These results agree with previous studies, further showing the close connection between hsa_circ_0007841 and its diagnostic and prognostic value in patients with MM.

Drug resistance is the one that causes the most trouble during the therapy of MM in clinic settings and need urgent solution (28, 29). In our study, hsa_circ_0007841 expression was also detected in drug-resistant cell lines (KM3/BTZ, U266/BTZ, 8226/BTZ) and MM patients. hsa_circ_0007841 also is the sponge molecule of hsa-miR-199a-3p. Fornari et al. found that miR-199a-3p regulated mTOR and c-Met to influence the doxorubicin sensitivity of human hepatocarcinoma cells (30). Lei et al. found that miR-199a-3p affected the multi-chemoresistance of osteosarcoma through targeting AK4 (31). Although, a large biological signal pathways of circRNAs involved in drug resistance are still unknown. More mechanisms and functions of chemoresistance-related circRNAs need to be further mined for advance of MM therapy, which may offer new approaches to reverse drug resistance.

Prognostic biomarkers can be used to assess the probability of disease recurrence and the prediction of clinical outcomes. Since prognostic indicators are not formulated by considering the factor of treatment, they can be used to guide individualized therapy. It has been shown that some genetic mutations are closely related to the poor outcome of MM, including chromosomal alterations *t* (4; 14), *t* (14; 16), and *t* (14; 20). Due to chromosomal translocation, oncogenes such as MMSET/FGFR3, CCND3, CCND1, MAF, and MAFB are still regulated by the IgH gene enhancer and their expressions increase (11, 32). As a result, the cyclin D family members are upregulated, leading to the dysregulation of the G1/S checkpoint. In the present study, hsa_circ_0007841 was closely related to chromosomal variations and genetic mutations in patients with MM. However, the present study is a single-center small-sample-size experiment, from which the results had limitations in guiding clinical treatment. In the future, the sample size should be enlarged. Further detection of its expression changes at different stages of disease progression is needed. Cell and animal experiments are needed to further explore its biological functions. Based on this fact, hsa_circ_0007841 may be used to predict the prognosis, recurrence, and drug resistance in patients with MM.

This study has evaluated the diagnostic and prognostic value of hsa_circ_0007841 as a circRNA in MM and the results provide the basis for preclinical research. Currently, the factors of age and complications are mainly considered for individualized therapy of MM, However, to further improve treatment outcome, molecular information will be needed to guide treatment. The endogenous competitive mechanism of circRNAs provides the theoretical basis for research on the replacement of antinucleotide chemotherapy. However, studies on circRNAs are still at the preliminary stage and very little has been hitherto understood about their functions and working mechanisms. But there is a high potential for clinical translational research with the advancement of research and development of RNA-targeted drugs based on circRNAs.

## Data Availability Statement

This manuscript contains previously unpublished data. The name of the repository and accession number(s) are not available.

## Ethics Statement

The studies involving human participants were reviewed and approved by 2016121. The patients/participants provided their written informed consent to participate in this study.

## Author Contributions

MG and CL designed and performed the study. HD and LG wrote the manuscript with inputs from all authors. YF and SJ performed the analytic calculations and statistical analysis. All authors provided critical feedback and helped to shape the research, analysis, and manuscript. We thank HX for her contribution to the collection of data.

### Conflict of Interest

The authors declare that the research was conducted in the absence of any commercial or financial relationships that could be construed as a potential conflict of interest.
