# Supplementary material for: hsa_circ_0007841: A Novel Potential Biomarker and Drug Resistance for Multiple Myeloma
Source: Front Oncol. 2019 Nov 19;9:1261. doi: 10.3389/fonc.2019.01261 (PMC6877741; doi:10.3389/fonc.2019.01261)
Supplement: Supplementary file 1 [file Table_1.DOCX]

Table S1：Characteristics of study population

| clinical characteristics | MM patients | value |
| --- | --- | --- |
| Sex |  |  |
|  | M | 53（62%） |
|  | F | 33（38%） |
| Age (yr) |  | Median 55（range：44-78） |
| Revised International Staging System |  |  |
|  | Stage 1 | 10（12%） |
|  | Stage 2 | 37（43%） |
|  | Stage 3 | 39（45%） |
| Isotype |  |  |
|  | IgG | 43（50%） |
|  | IgA | 22（26%） |
|  | Light chain | 12（14%） |
|  | Unclassified | 9（10%） |
| Percentage of myeloma cells in BM | |  |
|  | ＜40% | 57（66%） |
|  | ≥40% | 29（34%） |
| Bone disease |  |  |
|  | No | 7（8%） |
|  | Yes | 79（92%） |
| Renal insufficiency |  |  |
|  | No | 67（78%） |
|  | Yes | 19（22%） |
| Cytogenetic abnormality |  |  |
|  | No | 29（34%） |
|  | Yes | 57（66%） |
| Hemoglobin （g/dl） |  | 101.18±26.11 |
| Platelet count（×10^9^/L） |  | 169.29±88.43 |
| Neutrophil（×10^9^/L） |  | 2.81±1.96 |
| Albumin（g/L） |  | 34.21±6.46 |
| Globulin（g/L） |  | 32.90±22.40 |
| LDH（IU/L） |  | 265.23±161.23 |
| β2-MG（μg/ml） |  | 5.35±3.88 |
| creatinine（μmol/L） |  | 106.18±22.22 |
| Serum Calcium（mmol/L） |  | 2.21±0.20 |
